# Bilateral Spontaneous Pneumothorax in Chronic Silicosis: A Case Report

**DOI:** 10.1155/2014/561861

**Published:** 2014-03-16

**Authors:** Pritinanda Mishra, Sajini Elizabeth Jacob, Debdatta Basu, Manoj Kumar Panigrahi, Vishnukanth Govindaraj

**Affiliations:** ^1^Department of Pathology, Jawaharlal Institute of Postgraduate Medical Education and Research, Pondicherry 605006, India; ^2^Department of Pulmonary Medicine, Jawaharlal Institute of Postgraduate Medical Education and Research, Pondicherry, India

## Abstract

Silicosis is an occupational lung disease caused by inhalation of crystalline silica. People working in occupations like sandblasting, surface drilling, tunneling, silica flour milling, ceramic making, and so forth are predisposed to develop silicosis. Crystalline forms of silica are more fibrogenic than the amorphous forms, highlighting the importance of the physical form in pathogenesis. Lung biopsy is rarely performed for the diagnosis of silicosis as it can easily be detected by occupational history and radiological features. Patients with silicosis can develop complications like tuberculosis, lung cancer, progressive massive fibrosis, cor pulmonale, broncholithiasis, or tracheobronchial compression by lymph nodes. Pleural involvement in silicosis is rare. Spontaneous pneumothorax is a pleural complication that can develop in such patients. Usually in silicosis pneumothorax is unilateral. We hereby report the lung biopsy findings and discuss the mechanism of pneumothorax development in a case of chronic silicosis who, later on died during the course of the disease.

## 1. Introduction

Silicosis also known as “potters rot” is a form of pneumoconiosis caused by inhalation of crystalline silica. Crystalline silica is classified as a group 1 substance by the International Agency for Research on Cancer [1]. Currently the most prevalent chronic occupational lung disease in the world, silicosis, usually presents after decades of exposure as a slowly progressing nodular fibrosing pneumoconiosis. Pleural involvement in silicosis is rare and secondary spontaneous pneumothorax is the only described pleural complication [2]. In silicosis, pneumothorax is usually unilateral. We report the occurrence of bilateral spontaneous pneumothorax in a case of chronic silicosis.

## 2. Case Report

A 33-year-old male presented with progressive breathlessness and dry cough since the last 5 months and right sided pleuritic chest pain for 10 days. Patient was diagnosed elsewhere as miliary tuberculosis and was under antitubercular treatment (ATT) for four months. He was not a smoker. He worked as a bore-well driller for the past 10 years.

**Figure 1:**
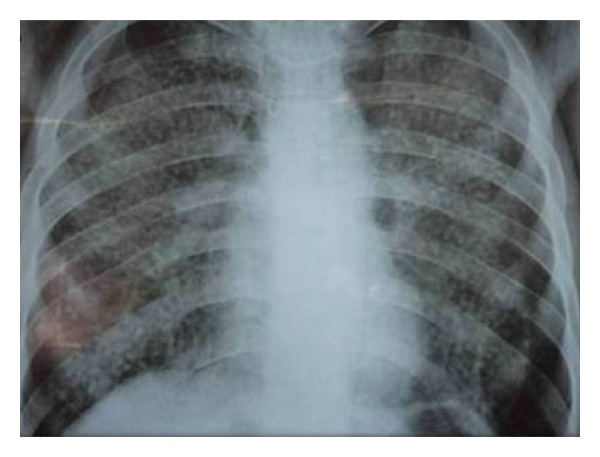
Chest X-ray: bilateral diffuse nodular opacities with bilateral pneumothorax.

Blood hemogram and renal and liver functions were normal. Admission chest radiograph showed bilateral, diffuse, well-defined large rounded nodular opacities with right secondary spontaneous pneumothorax (Figure 1).

Patient was managed with tube thoracostomy, supplemental oxygen, and analgesics. After three days patient complained of acute onset chest pain on left hemithorax and dyspnoea.

Chest radiograph revealed a new pneumothorax on left side for which immediate chest tube was placed. Subsequently, patient had symptomatic relief. Fibre optic bronchoscopy revealed normal airway. Bronchoalveolar lavage cytology showed only benign bronchial epithelial cells and smear and culture for* Mycobacterium tuberculosis* were negative. The diagnosis of military tuberculosis was questionable in view of larger size and nonresolution of nodules although four months of ATT and silicosis was more probable, considering his occupation. Hence, percutaneous biopsy of right lung was done that yielded a tiny tissue and showed only lymphoplasmacytic infiltrates. ATT was stopped and he was discharged after bilateral pleurodesis. Six months later he was brought to emergency with signs of severe respiratory failure. His condition deteriorated faster and despite adequate measures, he sustained a cardiopulmonary arrest and succumbed.

**Figure 2:**
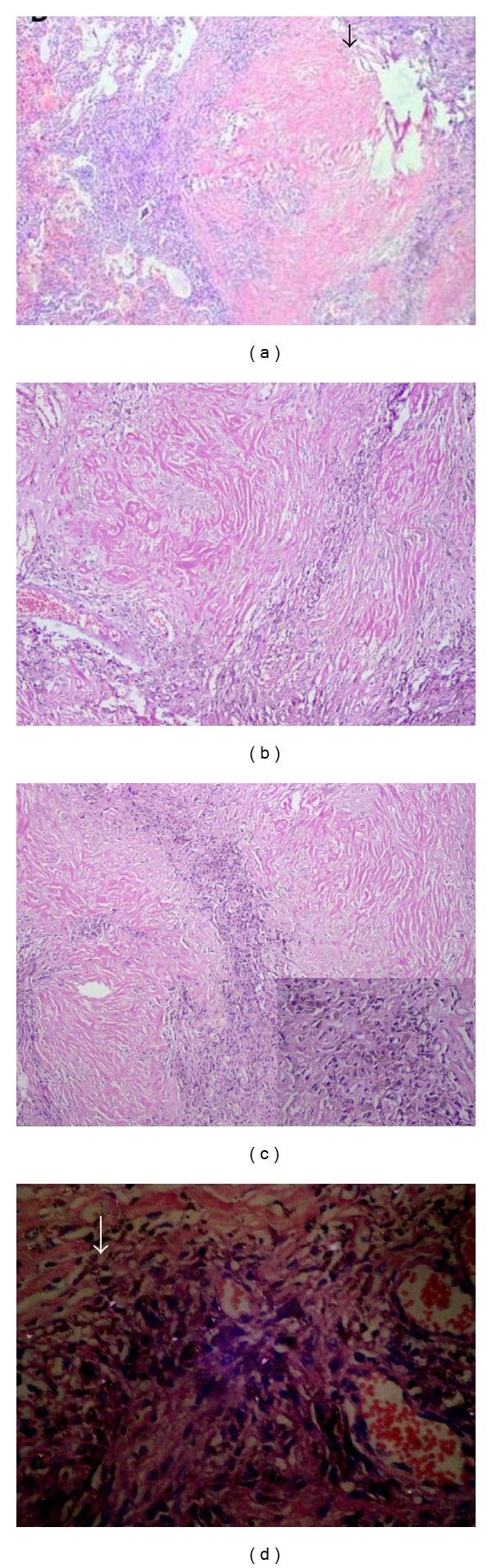
(a) Lung parenchyma showed collagenous nodules (arrow) (H&E ×100), (b) silicotic nodule: central zone of whorls of hyalinised fibrous tissue, midzone of concentrically arranged collagen fibres, and outer zone of randomly orientated collagen fibres and inflammatory cells (H&E ×200), (c) periphery of the nodules with dust laden macrophages and lymphoid cells (H&E ×200), inset: dust laden macrophages (H&E ×400), and (d) polarized light microscopy, white spots which represent silica crystals (white arrow).

Limited autopsy was done. Postmortem biopsy of bilateral lungs and liver was done. H and E sections of bilateral lungs revealed multiple collagenous nodules, some of which coalesced to form larger nodules (Figures 2(a) and 2(b)). The periphery of the nodules contained dust laden macrophages and inflammatory cells, predominantly lymphocytes (Figure 2(c)). There were no granulomas; acid fast staining was negative. Under polarized light microscopy, lung tissue showed white spots which represented silica crystals (Figure 2(d)).

## 3. Discussion

Workers most prone to develop silicosis are those involved in mining, tunneling, stonework, foundry work, sand blasting, and manufacture of ceramics. On the basis of dose and lag period since onset of exposure, silicotic lesions are classified as acute, accelerated, and chronic silicosis. Acute silicosis follows heavy exposure to very large amounts of silica and is characterized by rapid onset of severe dyspnoea cough, weakness, and weight loss, often leading to death. Accelerated silicosis occurs on short term exposure to large amounts of silica. Simple chronic silicosis follows long term exposure to low amounts of silica dust. Radiographically, small (<10 mm in diameter) opacities, typically rounded, can be seen in upper lung zones.

Inhaled silica is engulfed by alveolar macrophages that cannot digest it. Instead silica damages the alveolar lysosomal membranes leading to release of proteolytic enzymes into the cytoplasm and eventual death of the macrophage. Continued silica exposure leads to an alteration in macrophage function. There is release of inflammatory cytokines, for example, IL-1, free radicals, and growth factors [3, 4]. This stimulates collagen synthesis and production of antibodies against collagen. The anticollagen antibody stimulates the fibroblasts to produce more collagen that eventually leads to nodule formation. A primary feature that develops in lungs of silica exposed workers is nodule formation in the upper zones of the lung [5]. Nodule formation is usually the result of many years of exposure to relatively low levels of dust that contain silica quartz. A typical silicotic nodule has the following characteristics: central zone with whorls of dense, hyalinised fibrous tissue, a midzone with concentrically arranged collagen fibres similar to onion skinning, and an outer zone with randomly orientated collagen fibres, mixed with dust loaded macrophages and lymphoid cells. Under polarization microscopy, crystalline material can be observed as birefringent particles in the centre of the lesion [6].

Unilateral secondary spontaneous pneumothorax is the only described pleural complication in silicosis. Spontaneous pneumothorax is usually associated with chronic silicosis with progressive massive fibrosis. The involvement is unilateral in most cases. There are only very rare cases of bilateral involvement [7] and some of these bilateral involvements have been reported in accelerated silicosis also [8]. There are studies, where, in advanced silicosis secondary spontaneous pneumothorax is shown to be associated with the presence of bullae [9]. Due to direct toxic injury by silica, products of inflammatory response affect the elastic fibres of the alveolar wall leading to formation of bleb [8]. Massive fibrosis of lung results in a stiff nondistensible lung with an increased elastic recoil. Secondary spontaneous pneumothorax may be due to the rupture of the bullae and can be facilitated by the increased elastic recoil of lung parenchyma [9]. Some congenital alveolar defects and dysfunction of type II cells have also been considered to lead to development of pneumothorax [8].

Uncomplicated silicosis does not usually decrease life expectancy. Lung transplant has been performed in patients with end stage silicosis [10]. Rapid deterioration of the clinical condition of a patient with silicosis might be due to complications like progressive massive fibrosis, infection, cor pulmonale, pneumothorax, or tracheobronchial compression by lymph nodes. Occurrence of spontaneous pneumothorax, though rare in silicosis, should always be kept in mind as a complication in a patient with a known history of silicosis.

## References

[1] IARC Working Group on the Evaluation of Carcinogenic Risks to Humans (1997). *Silica, Some Silicates, Coal Dust and Para-Aramid Fibrils*.

[2] Fotedar S, Chaudhary D, Singhla V, Narang R (2010). Silicosis with bilateral spontaneous pneumothorax. *Lung India*.

[3] Rom WN (1991). Relationship of inflammatory cell cytokines to disease severity in individuals with occupational inorganic dust exposure. *American Journal of Industrial Medicine*.

[4] Saffiotti U, Daniel LN, Mao Y, Shi X, Williams AO, Kaighn ME (1994). Mechanisms of carcinogenesis by crystalline silica in relation to oxygen radicals. *Environmental Health Perspectives*.

[5] Boitsios G, Bankier AA, Eisenberg RL (2010). Diffuse pulmonary nodules. *American Journal of Roentgenology*.

[6] McDonald JW, Roggli VL (1995). Detection of silica particles in lung tissue by polarizing light microscopy. *Archives of Pathology and Laboratory Medicine*.

[7] Bairagya TD, Jana PK, Das SK, Bhattacharaya S, Dhua A (2012). Silicosis presenting with simultaneous bilateral spontaneous pneumothorax. *Annals of Tropical Medicine and Public Health*.

[8] Gupta KB, Manchanda M, Kaur P (2006). Bilateral spontaneous pneumothorax in silicosis. *The Indian Journal of Chest Diseases & Allied Sciences*.

[9] Mohebbi I, Hassani E, Salarilak S, Bahrami AR (2007). Do bullae and emphysema increase risk of pneumothorax in silicosis?. *Journal of Occupational Medicine and Toxicology*.

[10] Mao WJ, Chen JY, Zheng MF (2011). Lung transplantation for end-stage silicosis. *Journal of Occupational and Environmental Medicine*.

